# Activation of TRPM7 by naltriben enhances migration and invasion of glioblastoma cells

**DOI:** 10.18632/oncotarget.14496

**Published:** 2017-01-04

**Authors:** Raymond Wong, Ekaterina Turlova, Zhong-Ping Feng, James T. Rutka, Hong-Shuo Sun

**Affiliations:** ^1^ Department of Surgery, Faculty of Medicine, University of Toronto, Toronto, Canada; ^2^ Department of Physiology, Faculty of Medicine, University of Toronto, Toronto, Canada; ^3^ Department of Pharmacology, Faculty of Medicine, University of Toronto, Toronto, Canada; ^4^ Institute of Medical Science, Faculty of Medicine, University of Toronto, Toronto, Canada

**Keywords:** glioblastoma, U87, TRPM7, naltriben, migration

## Abstract

Glioblastoma (GBM), the most common and aggressive brain tumor in the central nervous system, remains a lethal diagnosis with a median survival of < 15 months. Aberrant expression of the TRPM7 channel has been linked to GBM functions. In this study, using the human GBM cell line U87, we evaluated the TRPM7 activator naltriben on GBM viability, migration, and invasiveness. First, using the whole-cell patch-clamp technique, we showed that naltriben enhanced the endogenous TRPM7-like current in U87 cells. In addition, with Fura-2 Ca^2+^ imaging, we observed robust Ca^2+^ influx following naltriben application. Naltriben significantly enhanced U87 cell migration and invasion (assessed with scratch wound assays, Matrigel invasion experiments, and MMP-2 protein expression), but not viability and proliferation (evaluated with MTT assays). Using Western immunoblots, we also detected the protein levels of p-Akt/t-Akt, and p-ERK1|2/t-ERK1|2. We found that naltriben enhanced the MAPK/ERK signaling pathway, but not the PI3k/Akt pathway. Therefore, potentiated TRPM7 activity contributes to the devastating migratory and invasive characteristics of GBM.

## INTRODUCTION

With a 5-year survival rate of < 10% (NEJM 352:987–996, 2005), glioblastoma (GBM) is a lethal progressive brain tumor. GBM unrestrictedly proliferates and invades throughout the brain tissue by degrading the extracellular matrix, contributing to its considerable treatment resistance [1]. Therapeutic options for GBM patients are extremely limited and with unsatisfactory outcomes [2]. The standard treatment is surgical resection of the tumor, followed by temozolomide and radiation [1]. However, due to the lack of specificity, the currently used adjuvants are highly cytotoxic [3]. Further research is necessary to elucidate the mechanisms underlying GBM functions in order to discover alternatives which kill tumors while sparing healthy cells. In essence, we need to better understand the involvement of the potential therapeutic targets for GBM if we hope to develop treatments that are highly specific to GBM with minimal toxicity.

Expressed ubiquitously in almost all tissues, TRPM7 is a non-specific divalent cation channel that serves as a Ca^2+^ conductor, and belongs to the melastatin subfamily within the transient receptor potential (TRP) ion channel superfamily. Ion channels comprise an important target class for drug development. TRPM7 has been indicated as a potential therapeutic target for GBM [4–7] in addition to other cancer types, including: pancreatic cancer, leukemia, head and neck cancer, prostate cancer, retinoblastoma, nasopharyngeal cancer, gastric cancer, ovarian cancer, and breast cancer [8–13]. Previous studies [4–7] have suggested that the involvement of TRPM7 in GBM is due to the channel's aberrant activity and up-regulation in the cancerous tissues by using techniques to reduce TRPM7 functions (i.e. knock down, or pharmacological inhibition). However, there is no report in the literature examining whether potentiation of TRPM7 channel activity would enhance GBM functions (i.e. proliferation, migration and invasion).

Until recently, reliable pharmacological compounds that can serve as specific TRPM7 agonists were unavailable. A set of 20 small molecules with various structural backbones that can induce TRPM7 currents was screened using a Ca^2+^ imaging-based assay [14]. Compared to the other compounds, the δ opioid antagonist naltriben was the most selective TRPM7 positive gating modulator. The EC_50_ of naltriben for TRPM7 was calculated to be ~20 μM; at 50 μM, naltriben had no effects on other TRP channels, including TRPM2, TRPM8 and TRPV1 [14]. In addition, the authors found that naltriben reversibly activated TRPM7 channels even in low PIP_2_ conditions and without intracellular Mg^2+^ depletion. Using a series of mutagenesis experiments, the authors also showed that the site of TRPM7 activation by naltriben resided in the TRP domain. Originally used as an antagonist of δ opioid receptors [15], it should be noted that although naltriben shows high structural similarity with other opioid receptor antagonists (e.g., naltrindole), none of the others can stimulate TRPM7 channels.

The current study evaluated the involvement of TRPM7 on GBM functions through a novel approach. That is, we assessed whether potentiating TRPM7 activity with naltriben would enhance U87 cell viability, migration and invasion. In addition, we explored the underlying potential mechanisms by studying the signaling pathways involved. We used MTT, scratch wound, and Matrigel invasion assays, combined with Western immunoblots, Ca^2+^ imaging, and patch-clamp electrophysiology to examine the effects of naltriben on the functional outcomes of U87 cells.

## RESULTS

### Endogenous TRPM7-like currents in U87 cells are potentiated by naltriben

**Figure 1:**
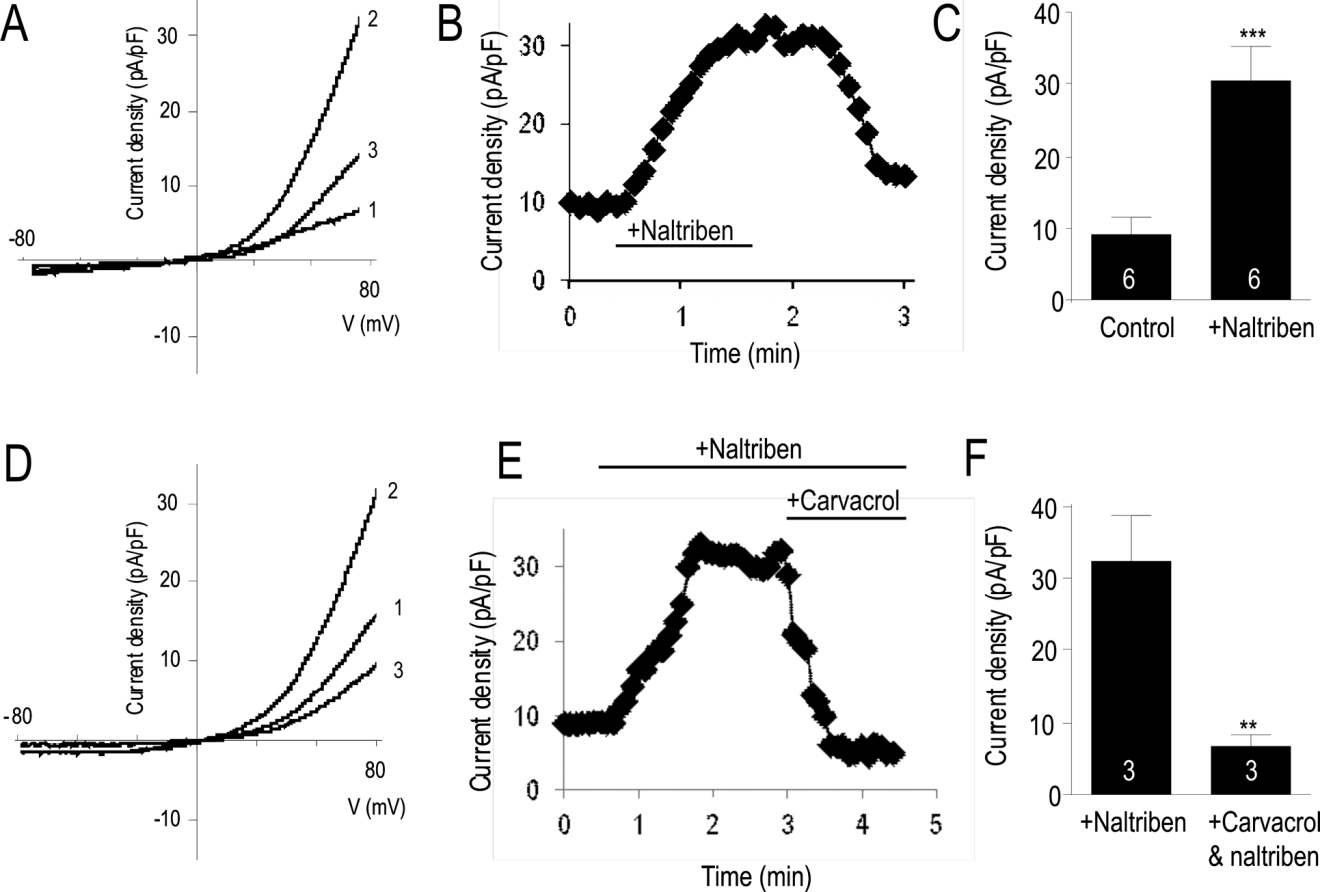
Naltriben activated TRPM7-like currents in U87 cells (**A**) Representative current density-voltage trace (1 is bath solution; 2 is perfusion of 50 μM naltriben, *note that maximal activation was observed ~1 min after naltriben application*; 3 is wash). (**B**) Representative time course of the outward current of TRPM7 at +80 mV. (**C**) Summary chart comparing the outward current at +80 mV between control versus activation of TRPM7 with naltriben. ***represents *p* < 0.001 (Student's *t-test*, *n* = 6/group). (**D**) Assessing the pharmacological inhibition of naltriben-potentiated TRPM7-like currents using carvacrol (300 μM). Representative current density-voltage trace (1 is bath solution; 2 is perfusion of 50 μM naltriben; 3 is simultaneous perfusion of both naltriben and 300 μM carvacrol). (**E**) Representative time course of the outward current of TRPM7 at +80 mV. (**F**) Summary chart comparing the naltriben-potentiated outward current at +80 mV before and after application of carvacrol. **represents *p* < 0.01 (Student's *t-test*, *n* = 3/group).

We have previously reported that the TRPM7 channel mRNA and protein expressions, and its activity are upregulated in the U87 cells compared to normal human astrocytes [4]. Activation of TRPM7 currents by naltriben have previously been confirmed in primary ventricular myocytes [16], as well as in HEK293 cells overexpressing TRPM7 [14]. In the current study, we first verified the potentiating effect of naltriben on the endogenous TRPM7-like currents in U87 cells by whole-cell patch-clamp recording. Figure 1A and 1B show that naltriben (50 μM) perfusion resulted in the activation of robust TRPM7-like currents. Consistent with the shape and characteristics of TRPM7, the naltriben-activated currents were large and rectified outwardly [4]. Activation by naltriben was readily reversible because the TRPM7-like current decreased with washout of naltriben. As Figure 1C shows, the initial outward current density at 100 mV was 9.7 ± 2.4 pA/pF, and naltriben significantly potentiated the TRPM7-like current to 31.3 ± 4.9 pA/pF (*p* < 0.001; *n* = 6). In our previous study [4], we reported that 300 μM carvacrol inhibits the basal U87 TRPM7-like currents. Here, in order to further verify that the effects of naltriben are mediated by TRPM7, we used carvacrol to examine whether it can also inhibit the naltriben-potentiated TRPM7-like currents in U87 cells. Figure 1D–1F illustrate that the TRPM7-like currents potentiated by naltriben are inhibited by carvacrol, which reduced the TRPM7-like current to 6.8 ± 1.7 pA/pF. Our results provide evidence that the endogenous TRPM7-like currents in U87 cells are sensitive to naltriben potentiation.

### Prolonged (24 h) naltriben-treatment is cytotoxic likely due to sustained Ca^2+^ influx

**Figure 2:**
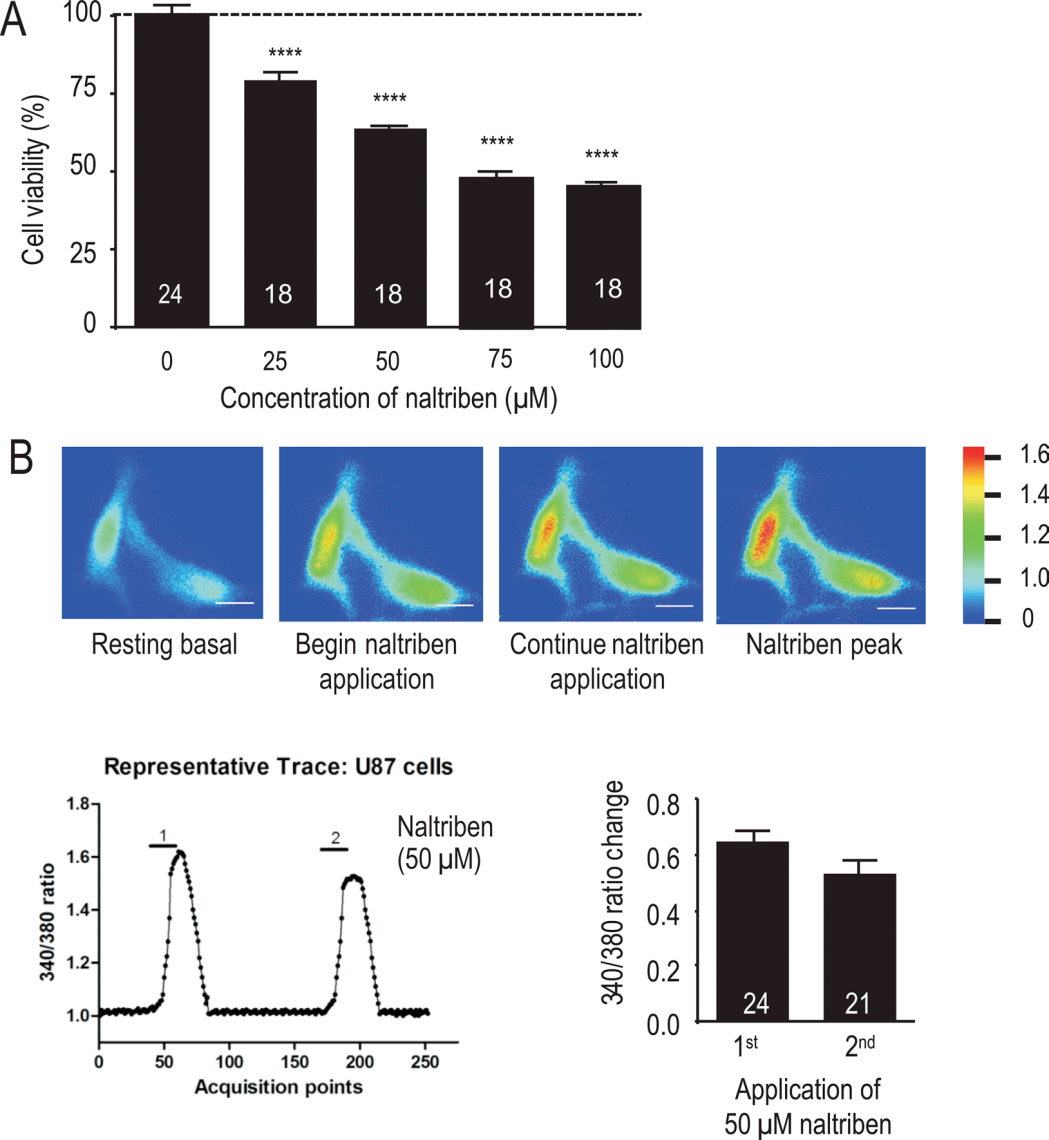
Naltriben induces Ca^2+^ influx, which might account for reduction in U87 cell viability (**A**) Summary chart of MTT assays, which were used to evaluate U87 cell viability. Cells were treated with vehicle (0.1% DMSO; control; *n* = 24) or with naltriben (25–100 μM; *n* = 18) for 24 h. **** represents *p* < 0.0001 (1-way ANOVA; compared with control). (**B**) Fura-2 ratiometric Ca^2+^ imaging experiments. U87 cells were pre-loaded with Fura-2 AM (2 μM) in the dark for 30 min at room temperature. Fura-2 Ca^2+^ signal was acquired at alternate excitation wavelengths of 340 and 380 nm. [Top] Representative raw images of U87 cells before (resting basal), during (start naltriben application; continue naltriben application), and after (naltriben peak) application of 50 μM naltriben. White scale bars represent 25 μm. Signal intensity color bar represents the 340/380 ratio. [Bottom Left] Representative 340/380 trace of Fura-2 ratiometric Ca2+ imaging experiments (1 is first application of 50 μM naltriben, 2 is second application of naltriben after wash). [Bottom Right] Summary chart of Fura-2 Ca^2+^ imaging experiments showing no significant difference between the Ca^2+^ responses of the initial exposure to naltriben (*n* = 24) and the subsequent perfusion following washout (*n* = 21).

Next, we assessed with the MTT assay whether naltriben affected U87 cell viability. Figure 2A shows that treatment with naltriben for 24 h reduced U87 cell viability in a dose-dependent manner (*p* < 0.0001; *n* = 18). This suggests a pathophysiological effect of prolonged TRPM7 activation due to sustained Ca^2+^ influx resulting in Ca^2+^ imbalance and ultimately cell death. The cytotoxic effects of Ca^2+^ imbalance are well documented [17–20], and cancer cells are not excepted [21–23]. To determine the effect of naltriben on TRPM7 Ca^2+^ dynamics, we performed Fura-2 ratiometric Ca^2+^ imaging on U87 cells, as described previously [24, 25]. As shown in Figure 2B, in the basal solution, the perfusion of U87 cells with 50 μM naltriben resulted in an increase of 340/380 ratio by 0.65 ± 0.04 (*n* = 24) from the baseline level. Subsequent perfusion of 50 μM naltriben after washout increased the ratio by 0.53 ± 0.05 (*n* = 21) from the baseline level, suggesting that there is no sensitization of TRPM7 Ca^2+^ current to repeated applications of naltriben. This illustrates that naltriben-treatment can elicit robust and sustained Ca^2+^ responses in U87 cells.

### U87 cell migration and invasion are enhanced by naltriben

**Figure 3:**
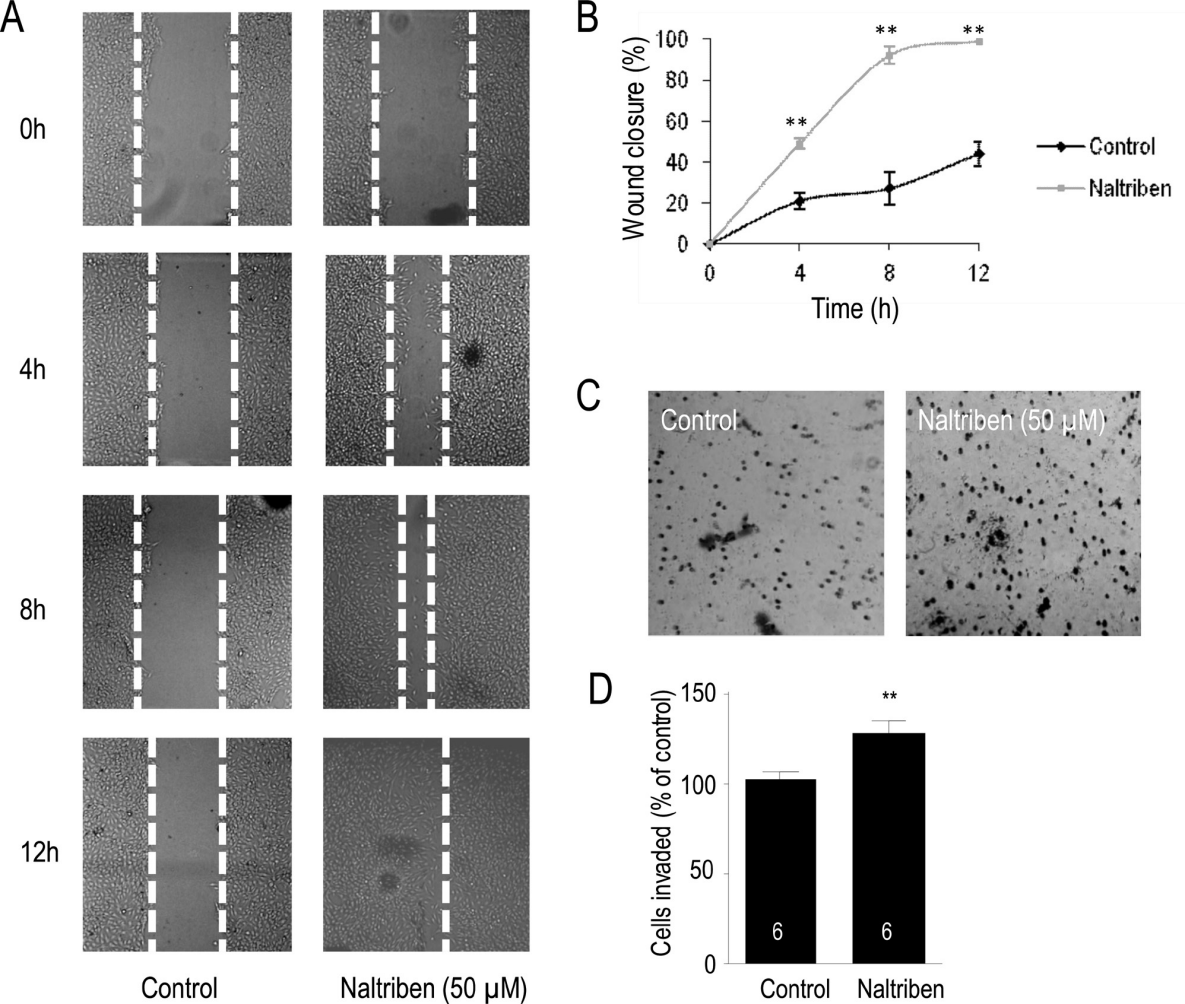
U87 cell migration and invasion were enhanced by naltriben (**A**) Representative images of wound healing following induction of scratch. Cells were treated with vehicle (0.1% DMSO; control; *n* = 6) or with naltriben (50 μM; *n* = 10), then images were captured at 0, 4, 8, and 12 hours, and gap closure was analyzed. (**B**) The wound closure of naltriben-treated cells at 4, 8, and 12 hours were significantly higher compared to the control group at the corresponding time points. ** represents *p* < 0.01 (Student's *t-test*). (**C**) Representative images from Corning Biocoat Matrigel invasion assays to detect cell invasion *in vitro*. Cells were treated with vehicle (0.1% DMSO; control; *n* = 6) or with naltriben (50 μM; *n* = 6) for 12 hours. (**D**) Invasion of naltriben-treated cells at 12 hours was significantly higher compared to the control group. ** represents *p* < 0.01 (Student's *t-test*).

The scratch wound assay and the Matrigel invasion assay were used to evaluate if naltriben-treatment had an effect on U87 cell migration and invasion, respectively. To assess cell migration *in vitro*, the scratch wound assay is a well documented procedure [26]. In Figure 3A and 3B, images of the migrating cells were captured at 0, 4, 8, and 12 hours after treatment with 50 μM naltriben, followed by analysis of the wound gap. In the control group, the wound closure was 21.2 ± 3.9%, 27.7 ± 8.1%, and 44.3 ± 5.9% (*n* = 6) at 4, 8, and 12 hours, respectively. This was slower than the wound closure in the naltriben-treated group at the corresponding time points: 49.1 ± 2.8%, 92.6 ± 4.3%, and 98.7 ± 0.2% (*p* < 0.01; *n* = 10). Our results indicate that naltriben significantly enhanced U87 cell migration. In Figure 3C and 3D, results from the Matrigel invasion assays show that U87 cell invasion after 12 h was significantly enhanced with naltriben (50 μM) treatment (89 ± 3 cells versus 127 ± 5 cells in the control and naltriben groups, respectively; *p* < 0.01; *n* = 6).

### Naltriben enhances MMP-2 as well as MAPK/ERK signaling, but not the PI3K/Akt pathway

**Figure 4:**
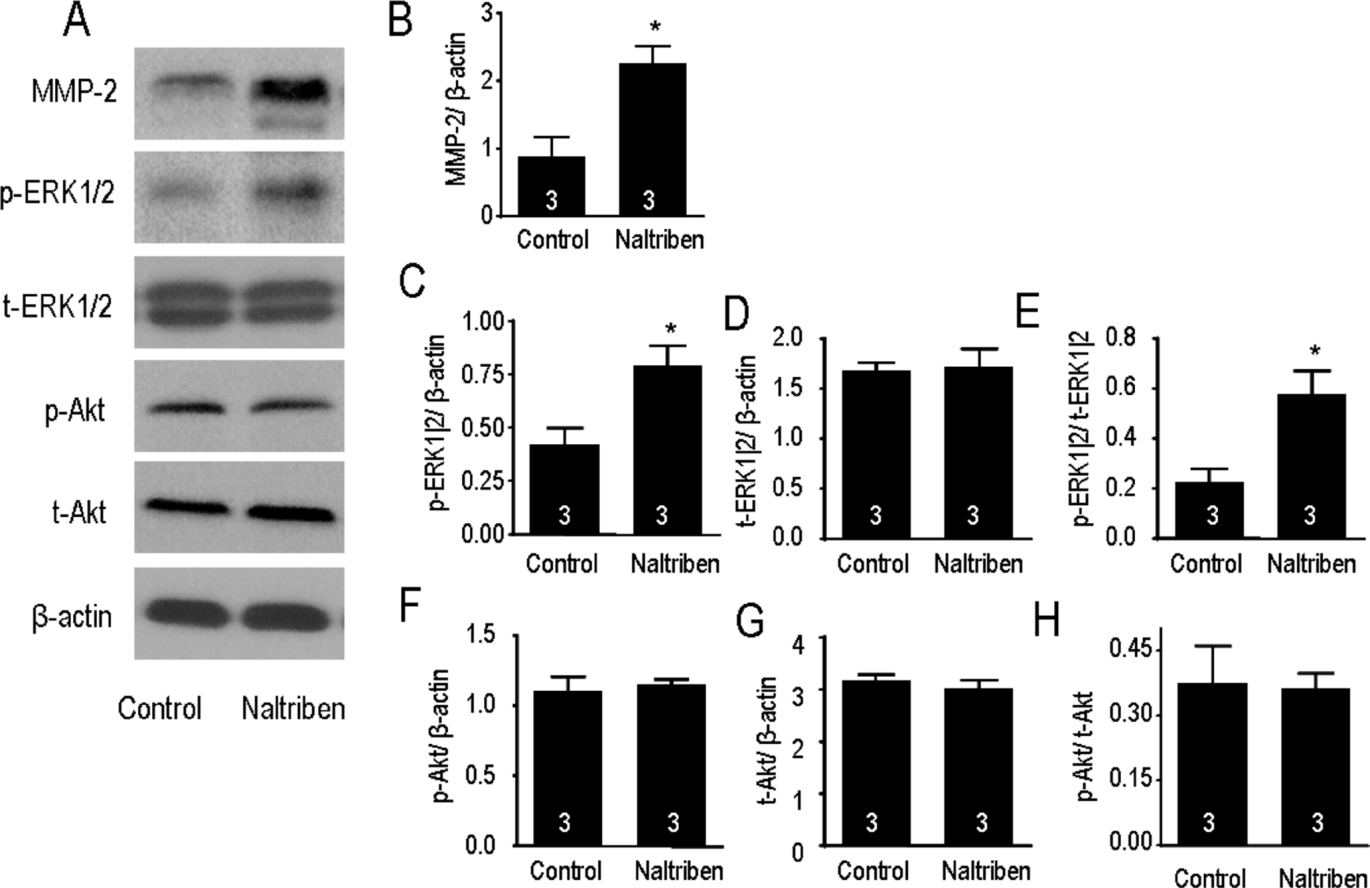
Naltriben upregulated the expression of MMP-2, and increased the phosphorylation of ERK1/2 in U87 cells (**A**) Representative images of Western immunoblotting. Cells were treated with naltriben (50 μM) for 24 h. Protein expression was then determined by Western immunoblot. Comparison between naltriben-treated cells and the control group was summarized in: (**B**) ratio of MMP-2/β-actin; (**C**) ratio of p-ERK1|2/β-actin; (**D**) ratio of t-ERK1|2/β-actin; (**E**) ratio of p-ERK1|2/t-ERK1|2; (**F**) ratio of p-Akt/β-actin; (**G**) ratio of t-Akt/β-actin; and (**H**) ratio of p-Akt/t-Akt. * represents *p* < 0.05 (Student's *t-test*, *n* = 3/group).

Previous *in vitro* and *in vivo* studies have reported greater migration and invasion in GBM with higher MMP-2 protein levels [1, 2, 27]. To assess whether naltriben enhances U87 migration and invasion via an MMP-2-dependent mechanism, we measured MMP-2 protein levels with Western immunoblots. As shown in Figure 4A and 4B, treatment of U87 with 50 μM naltriben for 24 h upregulated MMP-2 protein level (88.3 ± 28.2% versus 226.6 ± 25.1% in the control and naltriben groups, respectively; *p* < 0.05; *n* = 3).

The MAPK/ERK and PI3K/Akt signaling cascades are involved in many functions of GBM, including proliferation, migration, and invasion. Previously, we have shown that pharmacological block or knock-down of TRPM7 inhibited both pathways [4, 5]. In the current study, potentiation of TRPM7 by naltriben upregulated p-ERK1/2 protein levels (Figure 4A, 4C), but not p-Akt levels (Figure 4A, 4F). When normalized to β-actin levels, densitometry analysis shows that p-ERK1/2 levels (Figure 4C) are significantly increased by naltriben (42.2 ± 7.7% versus 79.5 ± 8.9% in the control and naltriben groups, respectively; *p* < 0.05; *n* = 3), while no change in t-ERK1/2 levels (Figure 4D). The p-ERK1|2/t-ERK1|2 ratio (Figure 4E) was greater in the naltriben group (22.7 ± 5.2% versus 57.8 ± 9.3% in the control and naltriben groups, respectively; *p* < 0.05; *n* = 3). On the contrary, naltriben-treatment had no effect on p-Akt levels (Figure 4F), t-Akt levels (Figure 4G), or the p-Akt/t-Akt ratio (Figure 4H). This is suggestive that MAPK/ERK, but not PI3K/Akt, signaling is involved in the naltriben-induced enhancement in U87 migration and invasion.

## DISCUSSION

In the current study, we report that naltriben: 1) potentiates endogenous TRPM7 channel activity and induces Ca^2+^ influx in the widely used U87 human glioblastoma (GBM) cell line; 2) enhances U87 migration and invasion; and 3) upregulates the MAPK/ERK signaling pathway.

Previously, we reported that the TRPM7 mRNA and protein, as well as channel activity are upregulated in the U87 cells compared to normal human astrocytes [4]. In the current study, we found that naltriben further augmented the TRPM7-like currents in U87 cells. The robust TRPM7 potentiation was stable until washout of naltriben, which was consistent with what other groups reported for TRPM7 overexpressed HEK293 cells or primary ventricular myocytes [14, 16]. For GBM, this is suggestive that although there is already an upregulation of basal TRPM7 activity compared to normal astrocytes, the channel can be further stimulated. The broader implication is that endogenous physiological or pathophysiological signaling pathways in GBM that positively modulate TRPM7 would enhance cancer migration and invasion. Ultimately, this would have devastating outcomes for the prognosis of patients with GBM.

Furthermore, we observed that the matrix metalloproteinase-2 (MMP-2) protein levels were increased when TRPM7 channel activity was potentiated by naltriben. This is consistent with our previous studies [4, 5], where we reported MMP-2 levels decreased following TRPM7 inhibition. In cancer cells, MMP-2 is localized in the invadosomes where it contributes to the degradation of the extracellular matrix, thereby assisting in the invasion process [28]. The present study showed that increasing TRPM7 activity enhanced GBM invasiveness potentially through MMP-2 upregulation, whereas our previous studies showed the vice-versa for TRPM7 inhibition [4, 5]. Taking our current findings in combination with our previous reports, there is strong evidence that TRPM7 activity regulates MMP-2 expression as the underlying mechanism linking the channel to the aggressive invasiveness of GBM.

Two important signaling cascades that play critical roles in proliferation, migration and invasion of GBM are the PI3K/Akt and MAPK/ERK pathways. Because these signaling pathways are overactive in many human cancers in addition to GBM [29–32], the discovery of effective Akt and ERK inhibitors has garnered attention. Currently, there are clinical trials evaluating drugs that inhibit the PI3K/Akt and MAPK/ERK signaling pathways [27]. Interestingly, patient response to treatments inhibiting only one of these signaling pathways is unsatisfactory [33]. This suggests that there is perhaps crosstalk between the PI3K/Akt and MAPK/ERK pathways, and a more effective strategy in the treatment of GBM would be to target an upstream regulator of both these pathways. Several studies showed that TRPM7 regulates the PI3K/Akt and MAPK/ERK pathways in a variety of cell types. Knock-down of TRPM7 in hepatic stellate cells prevented an increase in p-Akt and p-ERK1/2 levels following induction by PDGF-BB [34]. Additionally, silencing TRPM7 in OVCA cells or human lung fibroblast decreased the level of p-Akt, and in breast cancer cells, p-ERK1/2 was reduced [13, 35, 36]. In GBM, our lab previously showed that silencing or pharmacological inhibition of TRPM7 reduced the levels of p-Akt and p-ERK1/2, and consequently, downregulated the PI3K/Akt and MAPK/ERK pathways [4, 5].

**Figure 5:**
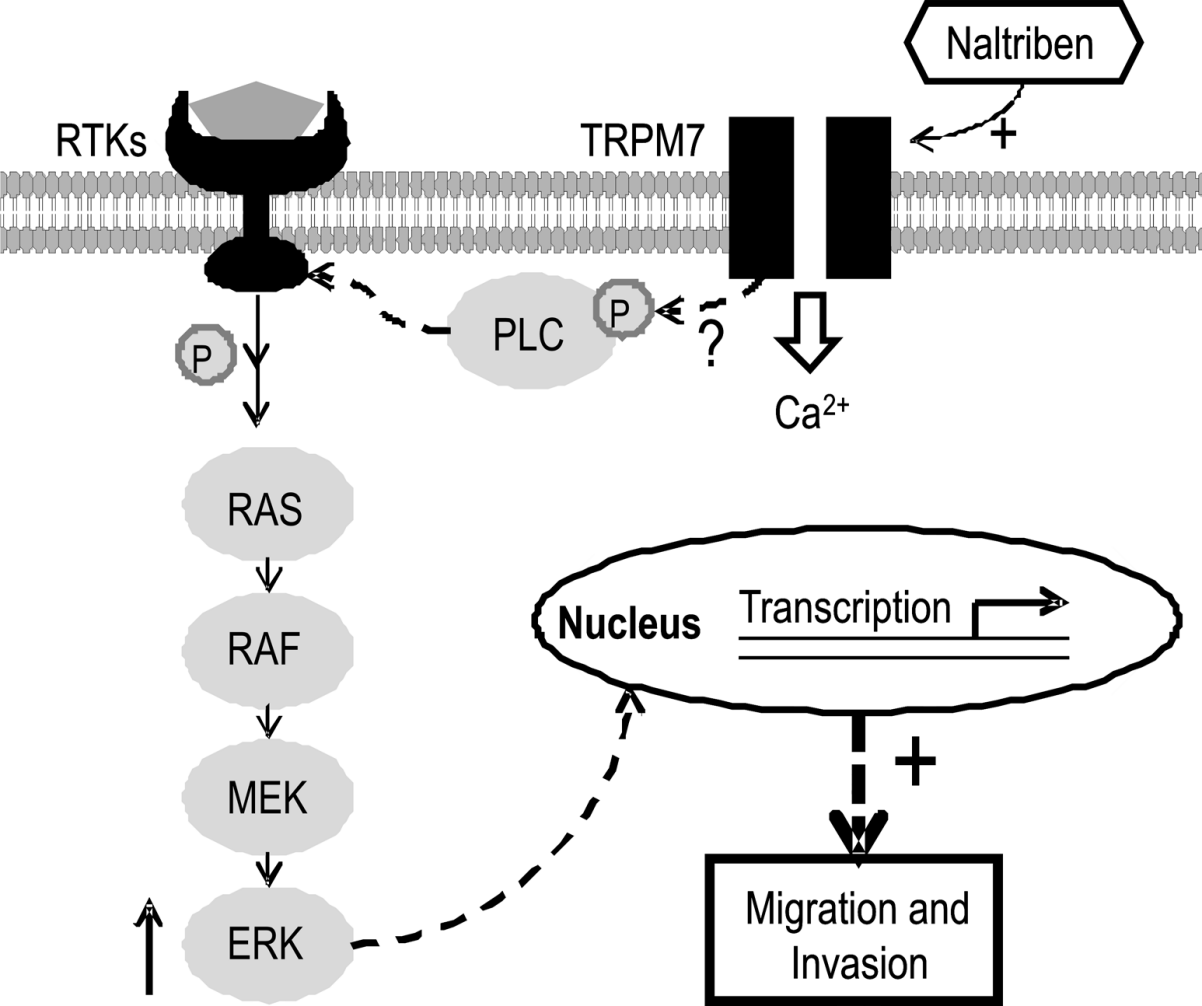
Schematic model illustrating the cellular signaling involved in the effects of TRPM7 potentiation on U87 migration and invasion The TRPM7 channel can respond to extracellular or cytosolic stimuli through regulation of Ca^2+^ and Mg^2+^ influx. In addition, TRPM7 contains a self-phosphorylating α-type Ser/Thr protein kinase domain that also modulates the phosphorylation of other cytosolic substrates such as PLC, which in turn, can regulate MAPK/ERK signaling. Consequently, functional gene transcription and translation are affected. Naltriben potentiates TRPM7, which upregulates MAPK/ERK signaling, and ultimately enhances U87 migration and invasion.

Therefore, based on these studies, we rationalized that potentiation of TRPM7 by naltriben would upregulate these pathways. However, in the present study, we found that although the expression of p-ERK increased, there was no change in the phosphorylation of Akt, which is a key protein of the PI3K/Akt signaling pathway. We have several explanations to account for this lack of change. Because the PI3K/Akt pathway can also be regulated by other ion channels aberrantly expressed in GBM, such as the TRPML-2 channel [37], potentiation in TRPM7 activity alone might not be sufficient to further upregulate the already excessive activation of PI3K/Akt. Furthermore, the main functional outcome of the overactive PI3K/Akt pathway in cancer cells is apoptosis prevention and proliferation promotion [38]. The unchanged PI3K/Akt signaling is consistent with our viability experiments, where we showed that potentiating TRPM7 with naltriben did not increase GBM viability. In fact, naltriben-treatment slightly decreased U87 viability in a dose-dependent manner, which we hypothesize to be due to sustained and prolonged (24 h) Ca^2+^ influx. This is based on the rationale that other groups have also shown that prolonged activation of TRPM channels can lead to Ca^2+^ imbalance resulting in cancer cell death [39–41]. The MAPK/ERK signaling pathway, on the other hand, has been shown to strongly contribute to the invasiveness of cancer cells in addition to proliferation [42]. Our current findings, where we saw increased GBM invasion alongside upregulated p-ERK levels following TRPM7 potentiation with naltriben, are consistent. Nonetheless, the molecular mechanism by which TRPM7 interacts with the MAPK/ERK pathway remains unclear. Because both MAPK/ERK and PI3K/Akt signaling can be regulated by phospholipase C [43], we speculate the involvement of TRPM7-associated PLC isozymes that interact with the TRPM7 α-type Ser/Thr protein kinase domain [44] (a schematic model is shown in Figure 5). Further investigation is necessary to elucidate this.

In summary, potentiation of TRPM7 channel activity with naltriben enhanced U87 migration and invasion, likely through upregulating MMP-2 protein expression as well as MAPK/ERK signaling. Our findings provide further evidence suggesting TRPM7 as a therapeutic target for GBM treatment.

## MATERIALS AND METHODS

### Reagents

Dulbecco's modified eagle's medium (DMEM), fetal bovine serum (FBS), and other cell culture materials were purchased from Gibco Life Technologies Corporation (USA). Anti-TRPM7 antibody (cat# ab85016) was purchased from Abcam (USA). Anti-MMP-2 (cat# 13132), anti-p-Akt (cat# 9271), anti-Akt (cat# 9272), and anti-p-ERK1/2 (cat# 5726) antibodies were obtained from Cell Signaling Technology (USA), while anti-ERK1/2 (cat# 442704) antibody was purchased from Millipore (Canada). Anti-β-actin antibody (cat# A1978) was purchased from Sigma-Aldrich (USA). Pierce BCA Protein Assay Kit was from Pierce Biotechnology (USA). Phenylmethylsulfonyl fluoride (PMSF), sodium chloride (NaCl), and sodium dodecyl sulfate (SDS) were obtained from Bioshop (Canada). All other reagents, unless specified, were from Sigma-Aldrich (USA).

### Cell culture

The U87 human GBM cell line was from the American Type Culture Collection (Manassas, VA). Cells were maintained on 10 cm culture dishes in Dulbecco's modified eagle's medium (DMEM) with supplements of 10% fetal bovine serum (FBS), and 100 U/mL streptomycin and penicillin at 37°C (5% CO_2_; 95% humidified air).

### Electrophysiology

Patch-clamp recording of TRPM7-like currents from U87 cells was done in the whole-cell configuration using an Axopatch 700B (Axon Instruments, Inc), similar to our previous study [4]. A 400 ms voltage ramp protocol from -100 to +100 mV (5 s interval) at 2 kHz (digitized at 5 kHz) was used. Data acquisition and analyses were done with the software pClamp 9.2 and Clampfit 9.2, respectively. All recordings were done at room temperature. The resistance of pipettes was between 5–10 MΩ after filling with pipette (intracellular) solution, which contained (in mM): 145 CsMSF, 8 NaCl, 10 HEPES, 10 EGTA, with the pH adjusted to 7.2 using CsOH. The bath (extracellular) solution had (in mM): 140 NaCl, 5 KCl, 2 CaCl_2_, 20 HEPES, 10 glucose, with pH adjusted to 7.4 and osmolarity to ~300 mOsm using NaOH and sucrose, respectively.

### Ca^2+^ imaging

A Fura-2 ratiometric Ca^2+^ imaging system as used to measure intracellular calcium ([Ca_2+_]_i_) as described previously [24, 25]. In the dark at room temperature, cells were preloaded with Fura-2 AM (2 μM) for 30 min. Using a Deltaram V single monochromator controlled by EasyRatioPro (PTI), Fura-2 Ca^2+^ signal was acquired at alternate excitation wavelengths of 340 and 380 nm. U87 cells were perfused with basal solution, which contained (in mM): 129 NaCl, 2 CaCl_2_, 1 MgCl_2_, 25 HEPES, 30 glucose, and 5 KCl (with pH adjusted to 7.3–7.4 and osmolarity to 320–330 mOsm), with or without 50 μM naltriben. An intensified charged-coupled device (ICCD) camera (PTI) digitized the signals. EasyRatioPro was used to calculate the fluorescence intensity (Poenie-Tsien) ratios of images.

### Cell viability and proliferation assay

MTT assays were carried out as previously described [4, 5]. In summary, U87 cells (5 × 10^4^ cells/mL) were cultured on 96-well plates and treated with naltriben at varying concentrations (25, 50, 75, or 100 μM) for 24 h, with the equivalent DMSO (0.1%; vehicle) as the control. Following treatment, MTT reagent (5 mg/ml MTT in PBS) was added at a dilution of 1:10 into each well. Cells were then incubated for three hours (37°C, 5% CO_2_). During incubation, mitochondrial enzymes reduce MTT (yellow) to form insoluble formazan (purple). Afterwards, the medium was discarded and 100 μL DMSO was added in each well to dissolve the formazan. Viability of cells is measured by quantifying the amount of formazan at an absorbance of 490 nm using a microplate reader (Syngery H1, Biotek, USA). U87 viability was expressed as a percentage of the 0.1% DMSO vehicle control. Three independent experiments were carried out, and each repeat consisted of eight (untreated cells) or six samples per group (naltriben-treated cells).

### Scratch wound assay

Cell migration was determined with scratch wound assays as previously described [4, 5]. In summary, U87 cells (5×10^4^ cells/mL) were cultured on 6-well plates, and when the confluency became > 90%, a scratch wound was created on the monolayer of cells with a 200 μL pipette tip. Cells were treated with either vehicle (0.1% DMSO; control) or 50 μM naltriben for up to 24 h. Images of the cells were taken from the same visual field throughout the experiment via a phase-contrast Olympus microscope (CKX41, ×10 objective). Quantification of wound closure was as follows: percentage of closure = Gap(T-T_0_)/GapT_0_*100% (where T = treatment duration; T_0_ = initial scratch).

### Matrigel invasion assay

U87 cell invasion was evaluated according to the manufacturer's instructions for the Corning Matrigel invasion chambers (8-μm polycarbonate Nucleopore filters, cat# 354480, BD Biosciences). After treating cells with vehicle (0.1% DMSO; control) or 50 μM naltriben for 24 h, 100 μL of cells (2.5 × 10^4^ cells/mL) in FBS-free DMEM was added to the top chamber. The bottom chamber contained 600 μL of complete medium to serve as a chemoattractant. Cells that are invading would degrade the Matrigel and migrate to the lower membrane surface of the top chamber. Invaded cells were then fixed in 100% methanol and stained with Toluidine blue (1%). Images were then captured with a digital camera through an Olympus microscope (CKX41). Quantification of invading cells was with the ImageJ software.

### Western immunoblot

Western immunoblots were conducted as previously described [4, 5]. In summary, cells were harvested in RIPA buffer containing a proteinase inhibitor cocktail (in mM: 50 Tris, 150 NaCl, 1 EDTA, 1 PMSF, 1 Na_3_VO_4_, 1 NaF; and 1% Triton X-100, 0.1% SDS, 1% C_24_H_39_NaO_4_, 1 μg/ mL aprotinin, 1 μg/mL leupeptin, 1 μg/mL pepstatin). The bicinchoninic acid (BCA) assay was used to measure the protein concentration of samples. Protein samples of equivalent amounts were then separated in an 8 or 12% SDS-PAGE gel, and transferred to a nitrocellulose membrane (Millipore, Billerica, MA, USA). Blocking was done with freshly prepared 5% milk, and then the membrane was immunoblotted overnight (4°C) with the following primary antibodies: anti-TRPM7 (1:1000), anti-p-Akt (1:1000), anti-Akt (1:1000), anti-p-ERK1/2 (1:1000), anti-ERK1/2 (1:1000), anti-MMP-2 (1:1000), and anti-β-actin (1:1000). This was followed by incubation for one hour at room temperature with the corresponding HRP-labeled secondary antibody (1:8000; Cell Signaling Technology, Danvers, MA, USA). The blots were developed with a chemiluminescence reagent kit (PerkinElmer Life Sciences Inc., Boston, MA, USA), and quantification via densitometry was carried out using the ImageJ software.

### Statistical analysis

To compare the difference between two groups, the Student's *t*-tests were used. For multiple comparisons, we used one-way ANOVA with subsequent Newman-Keuls test. Data are presented as the mean with SEM. *p* < 0.05 was regarded as statistically significant.
